# Core-Shell Magnetoactive PHB/Gelatin/Magnetite Composite Electrospun Scaffolds for Biomedical Applications

**DOI:** 10.3390/polym14030529

**Published:** 2022-01-28

**Authors:** Artyom S. Pryadko, Vladimir V. Botvin, Yulia R. Mukhortova, Igor Pariy, Dmitriy V. Wagner, Pavel P. Laktionov, Vera S. Chernonosova, Boris P. Chelobanov, Roman V. Chernozem, Maria A. Surmeneva, Andrei L. Kholkin, Roman A. Surmenev

**Affiliations:** 1 Physical Materials Science and Composite Materials Center, Research School of Chemistry and Applied Biomedical Sciences, National Research Tomsk Polytechnic University, 634050 Tomsk, Russia; vilajer@gmail.com (A.S.P.); phenics100@gmail.com (Y.R.M.); igor-parij1995@mail.ru (I.P.); bigbbro@yandex.ru (R.V.C.); surmenevamaria@mail.ru (M.A.S.); 2 International Research and Development Center “Piezo- and Magnetoelectric Materials”, Research School of Chemistry and Applied Biomedical Sciences, National Research Tomsk Polytechnic University, 634050 Tomsk, Russia; v.v.botvin@gmail.com; 3 Faculty of Radiophysics, National Research Tomsk State University, 634050 Tomsk, Russia; wagner_dv@mail.ru; 4 Institute of Chemical Biology and Fundamental Medicine, Siberian Branch, Russian Academy of Sciences, 630090 Novosibirsk, Russia; lakt@niboch.nsc.ru (P.P.L.); vera_mal@niboch.nsc.ru (V.S.C.); boris.p.chelobanov@gmail.com (B.P.C.); 5 Laboratory of Molecular Medicine, Novosibirsk State University, 630090 Novosibirsk, Russia; 6 Department of Physics and CICECO-Aveiro Institute of Materials, University of Aveiro, 3810-193 Aveiro, Portugal

**Keywords:** magnetoactive scaffold, poly-3-hydroxybutyrate, magnetite, composite, core-shell structure

## Abstract

Novel hybrid magnetoactive composite scaffolds based on poly(3-hydroxybutyrate) (PHB), gelatin, and magnetite (Fe_3_O_4_) were fabricated by electrospinning. The morphology, structure, phase composition, and magnetic properties of composite scaffolds were studied. Fabrication procedures of PHB/gelatin and PHB/gelatin/Fe_3_O_4_ scaffolds resulted in the formation of both core-shell and ribbon-shaped structure of the fibers. In case of hybrid PHB/gelatin/Fe_3_O_4_ scaffolds submicron-sized Fe_3_O_4_ particles were observed in the surface layers of the fibers. The X-ray photoelectron spectroscopy results allowed the presence of gelatin on the fiber surface (N/C ratio–0.11) to be revealed. Incubation of the composite scaffolds in saline for 3 h decreased the amount of gelatin on the surface by more than ~75%. The differential scanning calorimetry results obtained for pure PHB scaffolds revealed a characteristic melting peak at 177.5 °C. The presence of gelatin in PHB/gelatin and PHB/gelatin/Fe_3_O_4_ scaffolds resulted in the decrease in melting temperature to 168–169 °C in comparison with pure PHB scaffolds due to the core-shell structure of the fibers. Hybrid scaffolds also demonstrated a decrease in crystallinity from 52.3% (PHB) to 16.9% (PHB/gelatin) and 9.2% (PHB/gelatin/Fe_3_O_4_). All the prepared scaffolds were non-toxic and saturation magnetization of the composite scaffolds with magnetite was 3.27 ± 0.22 emu/g, which makes them prospective candidates for usage in biomedical applications.

## 1. Introduction

Functional materials responsive to different stimuli are of great importance for the well-being of modern society. Among all sources, various chemical and physical stimuli are currently being intensively investigated. Physical stimuli including magnetic or electric fields and ultrasound exposure have been of an interest in a variety of biomedical applications since they can remotely trigger a specific function of a biological object [1]. Magneto-responsive materials, as one of the representatives of the field responsive materials, have wide applications mainly in biomedicine (drug delivery, tissue engineering, biosensors, active diagnosis) [2], coatings (smart textiles and fibers) [3], and microelectronics (actuators, electromechanics) [4]. Functional magnetoactive materials are typically composites of polymers and inorganic fillers [5]. In terms of biomedical applications, magnetic filler of magneto-responsive materials, magnetite (Fe_3_O_4_) and cobalt ferrite (CoFe_2_O_4_) are used since they have a high saturation magnetization [6]. The latter, however, is potentially toxic to living tissues, which limits its widespread use in medicine [7]. On the contrary, magnetite is biocompatible, has relatively simple synthetic preparation procedures, and can be collected via magnetic separation [8].

As polymer components of magneto-responsive materials, polyethylene glycols, polyacrylamides [9], polyurethanes [10], fluorinated polymers [11], polyesters [12], and other polymers are used. Among the mentioned polymers, polyhydroxybutyrate (PHB) is of particular interest due to its biocompatibility, piezoelectricity, and ability to biodegrade [13]. Its degradation inside the body occurs slowly, thus, materials made of PHB are able to support a long-term tissue regeneration [14]. Moreover, D-3-hydroxybutyric acid—a degradation product of PHB—is a natural constituent of human blood that is nontoxic to body fluids and cause no inflammatory effects [15]. For this reason, PHB can be used as a drug carrier for the gradual and controlled release of the loaded drugs as well as a scaffold for the tissue engineering applications [16,17]. It has been shown that PHB based scaffolds are very attractive, especially for bone tissue engineering applications due to their piezoelectric properties [18,19]. Piezoelectricity plays an important role in bone tissue regeneration [20]. Piezoelectric polymers are able to modulate cellular behavior via surface charges generated in response to mechanical deformation. Furthermore, PHB can be embedded with magnetic particles to fabricate smart magneto-responsive materials providing physical stimulation of cells under a magnetic field [21,22]. Magneto-responsive scaffolds morphology, shape and geometry can be altered by the changes in strength and direction of the magnetic field to provide structural alignment [23], mechanical stimulation [24], and stem cell differentiation [25]. It has been shown that magneto-responsive materials are able to stimulate cell growth, proliferation and differentiation [26,27]. The potential applications of magneto-responsive materials for biomedical and tissue engineering applications have been summarized in recent reviews [28,29,30].

One of the drawbacks of PHB-based materials is the surface hydrophobicity. The hydrophobic properties of PHB, from the point of view of biomedical application, affect its controlled biodegradation, effective interactions with biological media, cells and different tissues [31,32]. The surface properties of PHB can be improved via various methods summarized elsewhere [13]. Among the methods known to increase the hydrophilicity of the PHB surface, the most promising is the preparation of blends with natural polymers [33], in particular, gelatin. Gelatin is a biodegradable and biocompatible polymer, which is in further hydrophilic, and non-antigenic. It also reveals plasticity and due to a combination of the properties mentioned, it is widely used in a variety of biomedical applications [34].

In the case of hybrid PHB-based materials, PHB/Fe_3_O_4_ and PHB/gelatin composite scaffolds have been well studied [22,35,36,37]. They reveal magnetic properties and improved hydrophobicity, yet to the best of our knowledge, there have been no attempts yet to fabricate magnetoactive materials based on PHB, gelatin, and Fe_3_O_4_. Thus, this work is devoted both to the fabrication of hybrid magneto-responsive PHB scaffolds doped with gelatin as well as doped with both gelatin and Fe_3_O_4_ and a comprehensive study of their structure, morphology, thermal, magnetic, and biological properties as prospective materials for a variety of biomedical applications.

## 2. Materials and Methods

### 2.1. Materials

Ferric (III) chloride hexahydrate (FeCl_3_-6H_2_O, Sigma-Aldrich, Steinheim, Germany), ferric (II) sulfate heptahydrate (FeSO_4_-7H_2_O, Sigma-Aldrich, Steinheim, Germany), 1,1,1,3,3,3-hexafluoroisopropanol (HFIP, Saint Louis, MO, USA), urea ((NH_2_)_2_CO, Sigma-Aldrich, Steinheim, Germany), Poly(3-hydroxybutyrate) (PHB, natural origin, MW 500,000) and gelatin (from porcine skin, type A) were purchased from Merck (Sigma-Aldrich, Steinheim, Germany). Deionized water obtained by Millipore Milli-Q system (Darmstadt, Germany) was used in all experiments.

### 2.2. Preparation and Characterization of Electrospun PHB/Gelatin/Magnetite Composite Scaffolds

#### 2.2.1. Synthesis of Magnetite Particles

Micron-sized Fe_3_O_4_ particles were synthesized by co-precipitation method. Briefly, 3.378 g of ferric (III) chloride hexahydrate, 1.713 g of ferric (II) sulfate heptahydrate and 6 g urea were loaded into a three-necked flask with a connected reflux condenser. Then, 50 mL of deionized water was added with constant stirring on a magnetic stirrer at a speed of 300 rpm for 10 min. The solution was then heated to 115 °C for 18 h with stirring at 800 rpm and then cooled to room temperature. The precipitate was separated by magnetic separation and decanted with deionized water to neutral pH. The samples were dried at 35 °C in a convection oven for two days.

#### 2.2.2. Electrospinning of Composite Fibrous Scaffolds

For PHB based scaffolds, dry PHB polymer powder was dissolved in hexafluorisopropanol (HFIP) for 24 h to achieve the concentration of 5% (*w*/*v*) and then used as a control. For PHB/gelatin composites, 5% (*w*/*v*) PHB and 10% (*w*/*v*) gelatin solutions were used for electrospinning. For PHB/gelatin/Fe_3_O_4_ composite scaffolds, 8% of Fe_3_O_4_ particles by weight of polymer were dispersed in HFIP and ultrasonicated (Scientz-IID, Ningbo SCienta Biotechnology Co. Ltd., Ningbo, China) for 4 h at room temperature. Then, Fe_3_O_4_ particles were added to the PHB/gelatin solution and got mixed with a shaker for 24 h. Pure and composite PHB scaffolds were electrospun at the collector rotation speed of 200 rpm, voltage of 8–9 kV and solution flow rate of 0.9 mL/h.

### 2.3. Characterization of the Scaffolds

The morphology of the particles and electrospun fibrous scaffolds was examined by a Scanning Electron Microscope (SEM) (EVO 10, Carl Zeiss AG, Jena, Germany). The obtained SEM images were used to calculate the diameters of magnetite particles and fibers using ImageJ software (V. 1.8.0, National Institute of Health, Bethesda, MD, USA).

The effect of Fe_3_O_4_ particles on the crystal structure of PHB and composite scaffolds have been characterized by X-ray diffraction (XRD) using a XRD–6000 diffractometer (Shimadzu Corporation, Kyoto, Japan) with CuKα radiation (λ = 0.154 nm) in the 2θ range from 5° to 65° at a step size of 0.01°/2θ at 40 kV and 30 mA.

From the XRD patterns, the crystallite size *D* of the crystalline phase of the scaffolds in the direction perpendicular to the (020) and (110) crystallographic plane was calculated using the Scherrer equation [38]:

(1) $$D=\frac{k\lambda }{\beta cos\theta }$$

where *k* is proportionality constant, *λ* is the X-ray wavelength (nm), *β* is the enlargement of the measured diffraction line at mid-height of its maximum intensity (in radian unit), and *θ* is the XRD peak position. The proportionality constant *k* is a function of the geometrical shape of the crystal. When the geometry of the crystallites is not known, it is assumed to be spherical with *k* being a value of 0.9.

Raman spectra and optical photographs were recorded using NT MDT (NT-MDT Spectrum Instruments, Zelenograd, Russia) system equipped with a 100× objective. Excitation was performed with a 633 nm laser with a maximum power of 50 mW. To prevent heating and oxidation of the magnetite particles, the laser power was reduced to 5 mW.

To characterize the surface composition, X-ray photoelectron spectroscopy (XPS) was performed using a Thermo Fisher Scientific XPS NEXSA spectrometer (Thermo Fisher Scientific, Waltham, MA, USA) with a monochromated Al K_α_ Alpha X-ray source working at 1486.6 eV. The XPS survey spectra were acquired at the pass energy 200 (eV) and energy resolution 1 eV from the surface area of 400 µm^2^. The high-resolution spectra were acquired at the pass energy was 50 (eV) and energy resolution 0.1 (eV). The flood gun was used to compensate the charge. To estimate the amount of gelatin in the near-surface layer of the scaffold by the XPS method, a washing procedure was performed as follows. The scaffolds were incubated in 5 mL of saline for 2 h at 25 °C, then 2 h at 70 °C. The scaffolds were then washed with deionized water 5 times for 20 min. Incubation and washing were carried out with the constant stirring of liquids.

Differential scanning calorimetry (DSC) was performed on DSC Q2000 Instruments (TA Instruments, New Castle, DE, USA) at the range 50–250 °C in the nitrogen atmosphere at a heating rate of 10 °C/min. Crystallinity (X_c_) of PHB scaffold and its composite with gelatin and magnetite was evaluated using the following formula:

(2) $$X_{c}=\frac{{\Delta H}_{f}}{{\Delta H}_{f}^{0}}\cdot 100\%,$$

where a heat of fusion for 100% crystalline PHB (${\Delta H}_{f}^{0}$) equal 146 J∙g^−1^ [39].

The magnetic properties of electrospun composite scaffolds were investigated at a temperature of 300 K with an external pulsed magnetic field from 0 to 6.5 kOe using a vibrating sample magnetometer. The measurements were carried out according to the method described elsewhere [40].

The contact angle was measured on a Drop Shape Analyzer–DS A25 (Kruss GmbH, Hamburg, Germany) using water as a solvent (drop volume, 1 µL; shooting speed, 160 frames per second) and no less than 10 measurements per sample. Data presented as mean ± error of the mean.

### 2.4. Cell Cultivation and Determination of Scaffolds Cytotoxicity

HeLa cells and human gingival fibroblasts (GF) were cultured in IMDM medium (Gibco, Carlsbad, CA, USA) containing 10% fetal calf serum (Gibco, Carlsbad, CA, USA) and antibiotics penicillin and streptomycin (100 U/mL) in an atmosphere of 5% CO_2_ at 37 °C. To assess the cytotoxicity of the obtained materials, disks 10 mm in diameter (~0.785 cm^2^) were cut from the scaffolds using a custom-made device. The cytotoxicity of the materials was carried out according to ISO 10993-5:1999 «Biological evaluation of medical devices—Part 5: Tests for in vitro cytotoxicity» using extracts from the material and the method of direct contact. To determine cytotoxicity using extracts from the material, scaffolds (disks with a diameter of 10 mm) were incubated in IMDM medium for 24 or 48 h, and then cells cultured in 48 well plates were incubated with the obtained extracts for 24 or 48 h, correspondingly. To determine the cytotoxicity of the scaffolds by direct contact, they were placed in the wells of a 48-well plate and fixed with Teflon rings. The cells were cultured on the scaffolds for 24 and 48 h. To get a calibration curve for the AlamarBlue test, the cells were plated on a plastic plate at the rate of 100, 50, 25, and 12.5% of their number in the remaining wells, incubated in the presence of a medium that did not contact the materials. The medium was removed, the cells were washed, and the IMDM medium without phenol red, containing 10% AlamarBlue dye, was added to them, incubated for 2–8 h at 37 °C and 5% CO_2_, then the supernatant was transferred into 96-well plates and the optical density of the solution was measured at wavelength 570 nm and reference wavelength 620 nm on a Multiskan GO spectrophotometer (ThermoScientific, Waltham, MA, USA). Using Microsoft Excel (V. 2016, Microsoft, Redmond, WA, USA), the dependence of optical density from the number of planted cells was calculated. Then, using this formula, the relative percentage of viable cells for each individual disc was calculated, the average value was found and a subsequent diagram was built. The cells on scaffolds were fixed with a 4% formalin at 4 °C for 24 h. Fixed cells rinsed with PBS, dehydrated using a graded ethanol series (50, 70, 80, 90, 96, and 100%) and then incubated in ethanol/hexamethyldisilazane (HMDS) solution (in a ratio at 1:1) followed by incubation in 100% HMDS. The samples were fixed on a sample stand using double-sided carbon tape and dried in air. The samples were sputter coated with 10 nm gold/palladium in SC7620 Mini Sputter Coater (Quorum Technologies, Laughton, UK) and analyzed using a SEM EVO 10 (Carl Zeiss AG, Jena, Germany) at an accelerating voltage of 10 kV.

GraphPad Prism software (V. 9.3.1, GraphPad Software, San Diego, CA, USA) was employed to analyze the statistical significance of the toxicity data and presented as described in [41]. Two-way analyses of variance (ANOVA) with Tukey’s multiple comparison tests were thus applied. The entire determination values are expressed as means ± SD, and they were significantly considered at *p*-value < 0.05, where *n* = 3.

## 3. Results

### 3.1. Study of the Morphology, Structure and Physico-Chemical Properties of Pure and Composite PHB Scaffolds

The morphology and phase composition of synthesized Fe_3_O_4_ were studied by SEM, XRD, and Raman spectroscopy (Supplementary Materials, Figures S1 and S2). Synthesized Fe_3_O_4_ particles have a submicron size of 329 ± 70 nm (Supporting Information, Figure S1). The XRD patterns of Fe_3_O_4_ (Supporting Information, Figure S2A) particles contain characteristic reflexes of magnetite at 2 Theta of 18.23, 30.0, 35.36, 42.99, 53.37, 56.87, and 62.45 corresponding to d_hkl_ crystal planes at (111), (220), (311), (400), (422), (511), and (440), respectively. The crystallite size calculated by the Scherrer equation was 36.1 nm. Raman spectrum (Supporting Information, Figure S2B) of Fe_3_O_4_ particles confirms the magnetite structure due to the presence of the peaks at 296, 533, and 667 cm^−1^.

**Figure 1:**
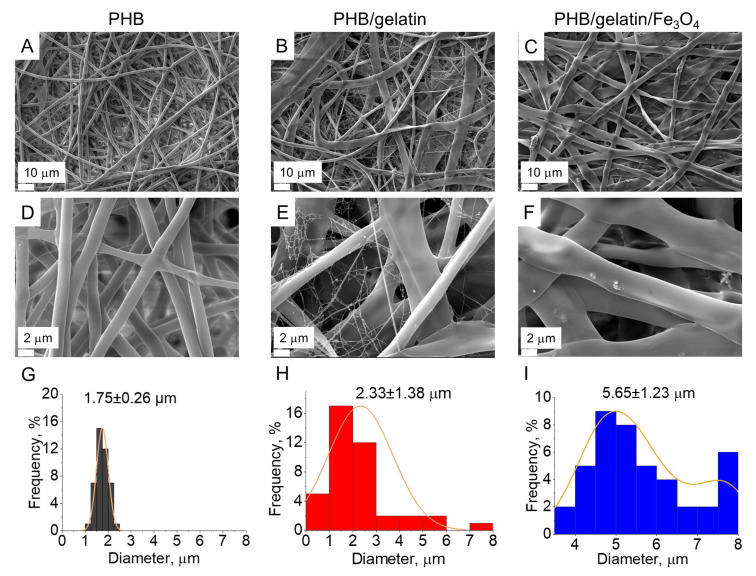
SEM images (**A**–**F**) and relative fiber diameter distributions (**G**–**I**) of the pure and hybrid scaffolds.

The effect of Fe_3_O_4_ particles and gelatin on the morphology of electrospun fibers was investigated via SEM. SEM images (Figure 1A–F) showed randomly-oriented bead-free microfibers with an average diameter of 1.75 ± 0.26 μm, 2.33 ± 1.38 μm and 5.65 ± 1.23 for PHB, PHB/gelatin and PHB/gelatin/Fe_3_O_4_ scaffolds, respectively.

The morphology of the composite fibers (Figure 1B,C,E,F) reveals a ribbon-like geometry upon addition of gelatin. Similar observation of the changes in the fiber morphology is reported elsewhere [42]. The formation of ribbon-shaped fibers was attributed to the rapid evaporation of the solvent from the fiber matrix. In case of hybrid PHB/gelatin/Fe_3_O_4_ scaffolds (Figure 1C,F) small protrusions from the Fe_3_O_4_ particles situated close to the surface are observed. The addition of gelatin increased the fibers diameter of hybrid PHB/gelatin and PHB/gelatin/Fe_3_O_4_ scaffolds (Figure 1G–I), which is associated with the increased viscosity and surface tension of the polymer solution [36]. Moreover, the surface charge density of the polymer solution may also affect fiber diameter and morphology of the electrospun fibers [43]. Similar observation of the fiber diameter increase is also reported elsewhere [44].

**Figure 2:**
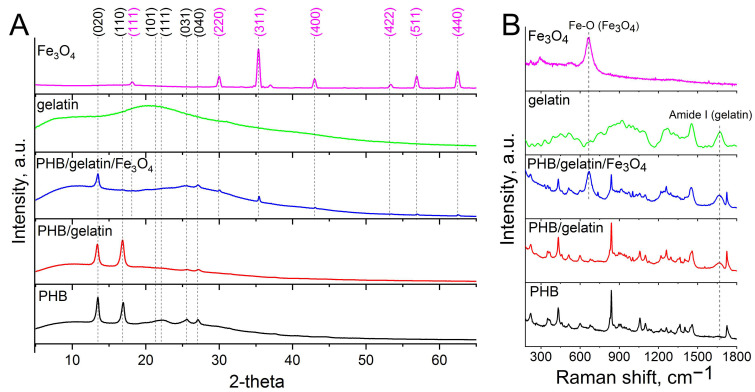
XRD patterns (**A**) and Raman spectra (**B**) of composite scaffolds.

Structure, phase composition and other properties of pure and composite scaffolds were studied by XRD, Raman spectroscopy, XPS, and DSC. Figure 2A shows the XRD results on the structure and phase composition of the electrospun scaffolds. The obtained diffraction patterns of PHB scaffolds clearly show the main characteristic peaks of the crystalline phase of PHB, observed at 2θ values of 13.6° (020) and 16.9° (110). The sample also contains the peaks at 21.4° (101), 22.4° (111), 25.5° (031/130), 26.9° (040) assigned to the planes of the α-phase of PHB (ICDD PDF card No. 00-068-1411). The pronounced peaks observed for hybrid PHB/gelatin/Fe_3_O_4_ scaffolds at 2θ 18.2° (111), 30.4° (220), 35.9° (311), 43.5° (400), 53.5° (422), 57.7° (511), 63.1° (440), which correspond to the magnetite with face-centered cubic lattice [45]. When blended and electrospun PHB along with gelatin, the XRD patterns indicated a decrease in the crystallite size (from 31 nm in PHB and 27 nm in PHB/gelatin to 21 nm in PHB/gelatin/Fe_3_O_4_ for (020) plane) of the obtained composite scaffolds [36].

Also, after the addition of the Fe_3_O_4_ particles in the polymer matrix, the intensity of the peak at 13.6° decreased, which was ascribed to inhibition of the polymer crystallization process caused by the magnetic particles decreasing the volume fraction of the crystalline phase in the fibers [35,46] (for more details please address to the DSC section of this study). Thus, submicron-sized Fe_3_O_4_ particles strongly affect the structure of PHB, changing the rate of polymer crystallization, polymer chains mobility and thus limiting the growth of lamellas [47].

Raman spectra of hybrid PHB/gelatin and PHB/gelatin/Fe_3_O_4_ scaffolds contain characteristic amide I peak at 1660 cm^−1^ and low intensity amide II peak at 1555 cm^−1^ confirming the presence of gelatin in the scaffolds (Figure 2B) [48]. The Raman spectra of PHB scaffolds exhibited the characteristic peaks presented in Table 1.

The spectrum of PHB/gelatin/Fe_3_O_4_ scaffold includes magnetite shift at ~670 cm^−1^ (Fe–O sym. str). Other less intensive peaks of magnetite at 540 cm^−1^ and 310 cm^−1^ are not observed in the composite scaffolds since they overlap with the reflexes of PHB.

**Figure 3:**
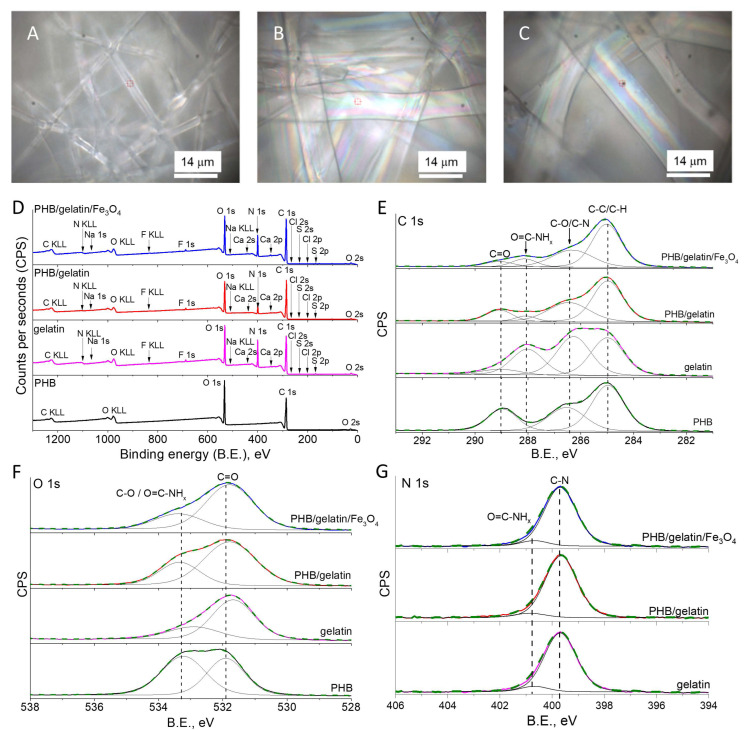
Optical microscope photographs (**A**–**C**) of pure PHB (**A**), PHB/gelatin (**B**) and PHB/gelatin/Fe_3_O_4_ scaffolds (**C**). Survey XPS spectra of the synthesized pure PHB, PHB/gelatin and PHB/gelatin/Fe_3_O_4_ scaffolds (**D**). High-resolution XPS spectra of C 1s, O 1s and N 1s regions for pure gelatin, pure PHB and composite scaffolds with addition of gelatin and Fe_3_O_4_ (**E**–**G**).

To reveal the changes in the surface composition of the PHB scaffolds after the addition of gelatin and Fe_3_O_4_ particles, XPS analysis was performed (Figure 3D). As observed, pure PHB scaffolds demonstrated the presence of C 1s and O 1s regions typical for PHB polymer [49]. In turn, the addition of gelatin in the polymer solution before electrospinning resulted in the presence of the pronounced peak of nitrogen associated with amine and amide group of gelatin [50], as well as a low intensity peaks of Na, S, F, Ca, and Cl elements most likely corresponding to salt contaminations of gelatin. It is worth mentioning that no iron was detected in the XPS spectra. At the same time, the optical and SEM images clearly demonstrated the presence of Fe_3_O_4_ particles inside the fibers (Figure 3C). Taking into account an XPS sensitive depth of up to 10 nm for polymers [51], Fe_3_O_4_ particles were inside the polymer fibers deeper than the sensitivity of the XPS analysis. In turn, the observed presence of N 1s can be explained by the formation of a gelatin thin coating on the surface of the fibers (Figure 3D).

Table 2 shows relative atomic concentrations of the detected carbon, oxygen and nitrogen, as well as salts mentioned as ‘others’ (F, S, Na, Ca, and Cl). As seen, the contribution of the salts was lower than 2% for synthesized pure gelatin and hybrid scaffolds. Additionally, the N/C ratio dropped twice for the composite scaffolds compared to the pure gelatin, most likely due to the contribution of the polymer. Therefore, this result indicates the presence of a very thin gelatin enriched layer on the fibers’ surface, since XPS sensitive depth varies up to 10 nm for polymer [51].

To verify this suggestion, the water treatment of the scaffolds with gelatin was performed after electrospinning (Supporting Information, Figure S3). As a result, the N/C ratio dropped from 0.10–0.11 to 0.03, thereby indicating the dissolution of a gelatin layer from the fibers surface (Table 2). Furthermore, other salts were not detected after composite scaffolds were immersed in saline solution. Additionally, the decrease in the intensity of O=C-CH peak at 288.1 eV from gelatin was observed in the high-resolution XPS spectra of the C 1s region for PHB/gelatin and PHB/gelatin/Fe_3_O_4_ scaffolds (Supporting Information, Figure S3B). At the same time, the intensity of a peak at 533.3 eV in O 1s region of hybrid scaffolds increased after exposure to saline solution in comparison with as-electrospun composite scaffolds, thereby indicating the dominant contribution from C-O group of PHB (Supporting Information, Figure S3C).

The analysis of high-resolution XPS spectra revealed chemical bonds on the surface of the scaffolds, as shown in Figure 3E–G. The C 1s region of PHB scaffolds was fitted with three typical peaks at 285 eV (C-C/C-H), 286.5 eV (C-O) and 289 eV (C=O) (Figure 3E) [49,52]. Taking into account the presence of the same functional groups, C 1s region of gelatin demonstrated the additional peak at 288.1 eV, which is assigned to O=C-CH groups [53,54,55]. Furthermore, in contrast to PHB, the higher intensity of the peak at 286.5 eV in C 1s region of gelatin is explained by the contribution of C-N groups [54]. In turn, C 1s regions of all hybrid PHB-based scaffolds with the addition of gelatin and Fe_3_O_4_ were deconvoluted with a pronounced presence of O=C-CH group. Therefore, the contribution of this functional group can be excluded from the peak at 533.3 eV in O 1s region of hybrid scaffolds [53], which demonstrated the reduced intensity of this peak in comparison with the C=O peak of pure PHB scaffolds (Figure 3F) [49]. Meanwhile, the N 1s region confirmed the presence of both O=C-CH group and C-N group in the hybrid scaffolds (Figure 3G) [53].

**Figure 4:**
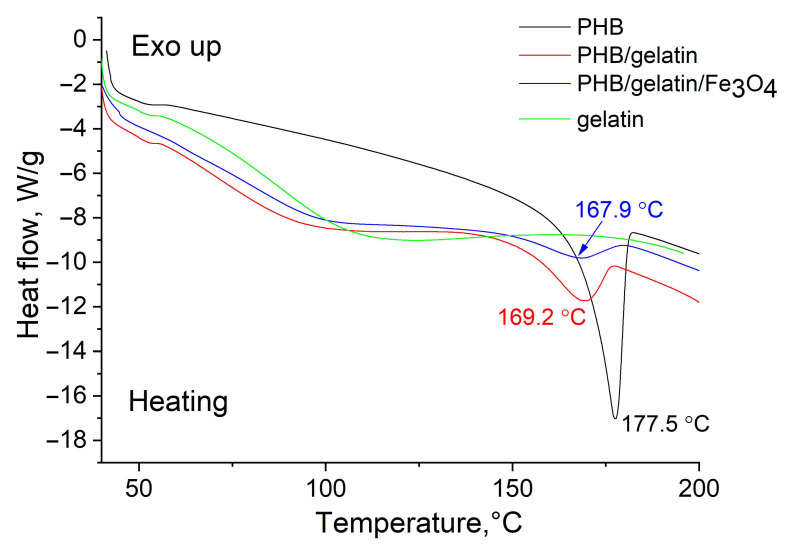
DSC curves obtained for pure gelatin as well as pure PHB, PHB/gelatin, PHB/gelatin/Fe_3_O_4_ scaffolds.

More details on the structure of the prepared scaffolds were revealed using a DSC analysis. The obtained DSC curves for pure PHB, PHB/gelatin, and PHB/gelatin/Fe_3_O_4_ scaffolds are presented in Figure 4. In the case of pure PHB scaffold, a characteristic endothermic peak of 177.5 °C corresponding to the melting temperature of the polymer is found [56]. The presence of gelatin in PHB/gelatin and PHB/gelatin/Fe_3_O_4_ scaffolds results in the decrease in melting temperature to 168–169 °C compared with pure PHB scaffold. It is reported that gelatin and PHB are thermodynamically immiscible polymers, therefore, phase separation occurs during elecrospinning leading to the formation of core-shell structure (PHB–core, gelatin–shell) [57]. Due to polyelectrolyte (polyampholyte) nature of gelatin, it tends to migrate in a similar manner as other polyelectrolytes towards the outer layer of the PHB/gelatin solution jet under the electrostatic repulsion forming shell layer [58]. Additionally, the interference observed in the optical images (Figure 3B,C) and XPS spectra of composite fibers with gelatin (Figure 3D–G) confirm the presence of a gelatin layer on the surface of PHB fibers. Therefore, the changes in melting temperature may be associated with the core-shell structure of the fibers affecting the heat transfer from the gelatin layer to the PHB core. The DSC curve of pure gelatin has no melting peak due to its amorphous nature. The curve includes a broad endothermic peak assigned as a denaturation temperature [59]. The obtained data of enthalpy of fusion and crystallinity are shown in Table 3.

Crystallinity of pure PHB scaffolds is in agreement with the data presented in the literature [56]. The composite PHB/gelatin and PHB/gelatin/Fe_3_O_4_ demonstrated a decrease in crystallinity. This tendency can be explained by the formation of a thin gelatin layer (shell), which prevents the crystallization of PHB (confinement effect). The incorporation of submicron Fe_3_O_4_ particles into the scaffolds also promotes a decrease in crystallinity of PHB.

Magnetic properties of the composites are affected by the phase composition, crystallite size and particles dimension [60]. It is known that the saturation magnetization increases with the increase in the particle and crystallite sizes. The saturation magnetization of magnetoactive composites, which reveals a positive effect on cell differentiation and tissue growth ranges from 2 to 5 emu/g [61,62,63]. For instance, poly(l-lactide)/Fe_3_O_4_ nanofibers exposed to a static magnetic field (SMF) of 100 mT possessed enhanced osteogenic differentiation of MC3T3-E1 cells compared to non-exposed ones [62]. In other work, electrospun poly(lactide-co-glycolide) scaffolds embedded with oleic acid-coated iron oxide nanoparticles were seeded with mouse pre-osteoblasts (MC3T3-E1 cell line) to investigate the effect of static magnetic field [61]. It has been shown that SMF exposure of 70–80 mT significantly improved cell attachment and osteogenic differentiation of mouse pre-osteoblasts as a result of the magnetically actuated mechanical stimuli induced through the nano-deformation of the magneto-responsive scaffolds.

**Figure 5:**
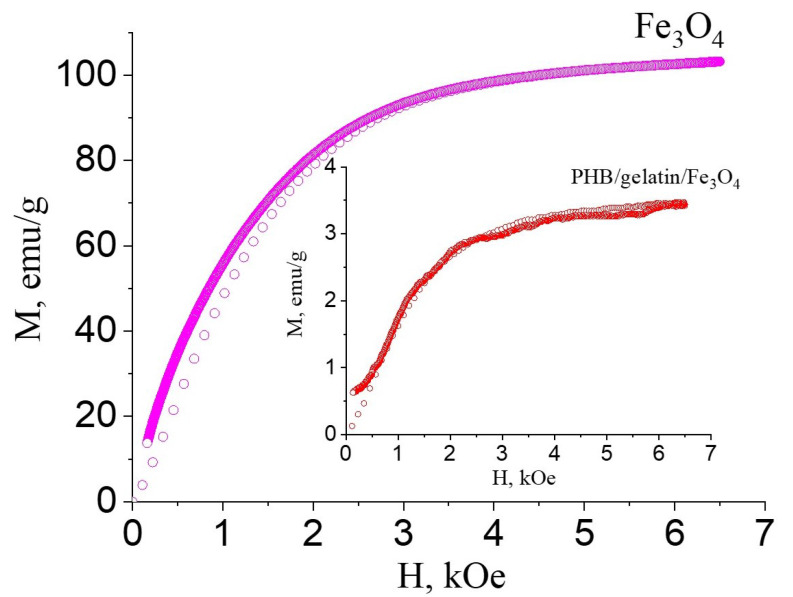
Magnetization curves of the Fe_3_O_4_ particles and composite PHB/gelatin/Fe_3_O_4_ scaffolds (inset image).

In order to estimate the prospective use of the developed scaffolds in medicine, the saturation magnetization values of the scaffolds was measured. The saturation magnetization for the composite PHB/gelatin/Fe_3_O_4_ scaffolds was 3.27 ± 0.22 emu/g (Figure 5, inset image), whereas saturation magnetization of submicron-sized Fe_3_O_4_ particles was 103 emu/g.

The saturation magnetization of magnetite particles in our research is a little higher than that reported in the literature [64] due to the presence in the samples of superparamagnetic particles (paraprocess) and the ordering of the magnetic moments of the surface layer, which possesses some structural defects. Saturation magnetization of the composite electrospun scaffolds obtained in this study is in the range of 2–5 emu/g reported in the literature for the different magnetoactive composites, thus, they can be potentially used for tissue engineering and regenerative medicine applications using external magnetic field [61,62,63].

### 3.2. Determination of Cytotoxicity of the Pure and Composite Scaffolds

**Figure 6:**
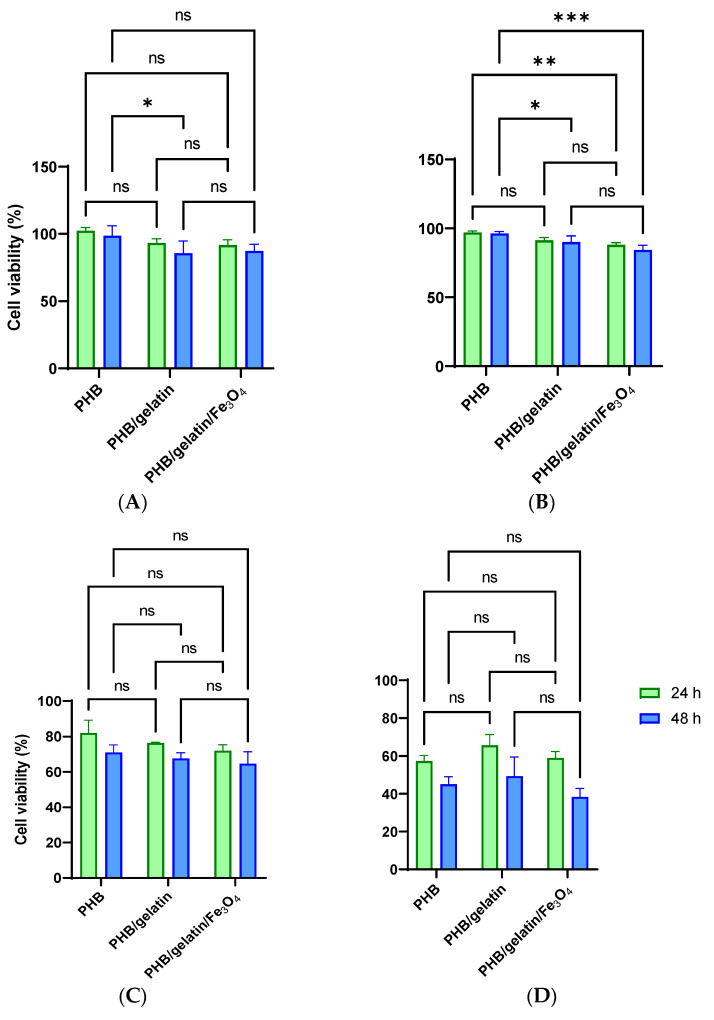
Cytotoxicity of pure PHB, PHB/gelatin, PHB/gelatin/Fe_3_O_4_ scaffolds for HeLa (**A**,**C**) and GF (**B**,**D**) cells. The data obtained in accordance to ISO 10993-5:1999 using extracts from the material (**A**,**B**) and the method of direct contact (**C**,**D**). Cells seeded in the same number on tissue culture plastic were used as a control (100%). Results are presented as means ± SD (*** *p* < 0.001, ** *p* < 0.01, * *p* < 0.05, and (ns) points to a non-significant difference).

The cytotoxicity of electrospun scaffold was evaluated by the extraction test (ISO 10993-5:1999) using primary and transformed cell lines and two time intervals (24 and 48 h). Longer incubation of the scaffolds in the culture medium provides better extraction of potentially toxic products, whereas a longer incubation of the extract with cells must increase the influence of potentially toxic substances on cell viability. It should also be mentioned that primary cells are usually more sensitive to toxic compounds or the culture medium composition that transformed the cells [65]. Human cervix adenocarcinoma HeLa cells and human primary fibroblasts demonstrated similar cellular responses to the extracts, which are not statistically different from that of the control scaffolds (PHB/gelatin compared with PHB/gelatin/Fe_3_O_4_). As such, the scaffolds can be considered as non-toxic to cells (Figure 6A). The statistically significant differences of PHB scaffolds from both PHB/gelatin and PHB/gelatin/Fe_3_O_4_ scaffolds, which are more prominent at 48 h of cell cultivation, is obviously connected to the release of gelatin from the scaffolds, as shown for similar materials elsewhere [66]. Gelatin added to the culture medium can interfere with cell adhesion, demonstrating more pronounced effect at longer incubation times. This effect is observed on HeLa and more sensitive GF cells although this is not connected with the toxicity of Fe_3_O_4_ particles.

**Figure 7:**
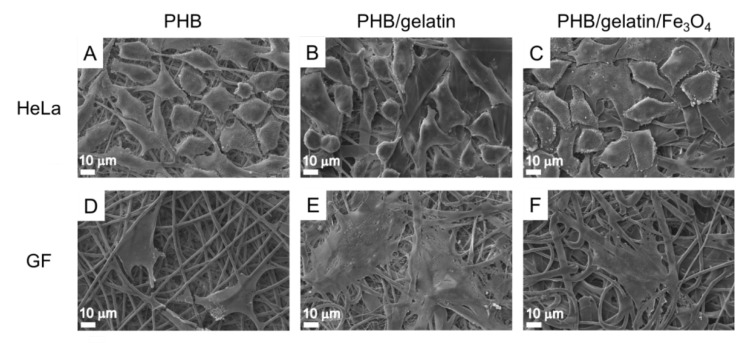
SEM images of HeLa (**A**–**C**) and GF (**D**–**F**) cell adhesion to PHB, PHB/gelatin, PHB/gelatin/Fe_3_O_4_ scaffolds.

This claim is supported by the cultivation of cells on the scaffolds, which demonstrated the absence of the toxicity independent on the scaffold composition. The number of HeLa cells adhered on the scaffolds surface almost did not depend on their composition, whereas GF cells were more susceptible to scaffolds composition and adhered better to PHB/gelatin ones. It should be mentioned that gelatin was removed partially from the fibers while being washed in saline solution, however residual quantities of gelatin nevertheless still supported GF cells adhesion. Actually, surface associated proteins can leave the fibers if they are not fixed by bifunctional reagents as well as crosslinking of proteins exposed at the fiber surface by bifunctional reagents, not only prevents their loss but promotes the increase in cell adhesion. The decrease in protein motility can be the reason of better cell adhesion [66,67]. HeLa cells adhered well to all types of scaffold, however the cells were spread better on the surface of PHB/gelatin as compared to other materials (Figure 7B). GF cells also spread better on PHB/gelatin matrices, forming multiple outgrowths probably due to the presence of a small diameter of the fibers in scaffolds. Despite the fact the XPS data demonstrated the absence of direct exposure of magnetite on the fiber surface and the results of better hydrophilicity of such scaffolds, HeLa cells have more pronounced granular boundaries on matrices with magnetite (Figure 7C). The contact angle decreased in PHB, PHB/gelatin and PHB/gelatin/Fe_3_O_4_ scaffolds from 92.14 ± 2.21° to 85.94 ± 3.95° and 81.77 ± 3.23°, respectively. Perhaps this phenomenon is associated with the poorer protein adsorption on hydrophilic surfaces [68,69], which leads to a minor decrease in cell adhesion to PHB/magnetite scaffolds. It should also be mentioned that, along with the chemical composition, the roughness and porosity of the matrices are the factors that determine the interaction of cells with scaffolds, namely, cell adhesion, proliferation, and migration [70,71]. The roughness within the range of 10–135 nm insignificantly influences the cell ability to attach to the matrix surface although a roughness exceeding 287 nm is rather inappropriate for cells to attach to [72,73]. Another factor that determines the colonization of scaffolds is the pore size, which influences the mechanism of cell–matrix interaction [74] and has had a stronger effect than the fiber diameter on the proliferation of dermal fibroblasts [75]. An insignificantly less efficient adhesion of GF cells toward the hybrid PHB/gelatin/Fe_3_O_4_ scaffolds (Figure 6 and Figure 7) may be explained not only by the chemical composition of the materials, but by considering the flat fibers are located further apart from each other when compared with other fibrous scaffolds (Figure 1).

The interaction of cells with gelatin enriched scaffolds could be increased further via treatment with glutaraldehyde, which allows a decrease in the molecular mobility of the surface exposed gelatin and thus provides better cell adhesion, as reported elsewhere [67]. Thus, the results presented demonstrated biocompatibility of the developed PHB-based scaffolds and the possibility of their use in the different biological systems.

## 4. Conclusions

Hybrid electrospun PHB/gelatin and PHB/gelatin/Fe_3_O_4_ and pure PHB scaffolds were synthesized and their morphology, structure, phase composition, thermal, magnetic, and biological properties were studied. The obtained scaffolds showed randomly oriented microfibers with an average diameter 1.75 ± 0.26 μm, 2.33 ± 1.38 μm and 5.65 ± 1.23 μm for PHB, PHB/gelatin and PHB/gelatin/Fe_3_O_4_ scaffolds, respectively. The incorporation of gelatin to PHB composites resulted in the formation of core-shell fibers (PHB–core, gelatin–shell) due to phase separation (thermodynamic immiscibility of both polymers) and its polyelectrolyte nature. The core-shell morphology of the fabricated composite scaffolds was confirmed by XPS, DSC and optical microscopy. The formation of core-shell structure in the case of PHB/gelatin and PHB/gelatin/Fe_3_O_4_ scaffolds resulted in a decrease in crystallinity (and crystallite size) of the PHB phase from 52.3% (31 nm) in pure PHB to 16.9% (27 nm) in PHB/gelatin and 9.2% (21 nm) in PHB/gelatin/Fe_3_O_4_ scaffolds due to the confinement effect. Saturation magnetization of PHB/gelatin/Fe_3_O_4_ scaffolds 3.27 ± 0.22 emu/g was in the range of 2–5 emu/g, as reported in the literature, as an optimal for magnetoactive materials. The cytotoxicity study of electrospun scaffolds demonstrated that both human cervix adenocarcinoma HeLa cells and human primary fibroblasts revealed a similar cellular response to the extracts, which were not statistically different from the control and thus allow scaffolds to be considered non-toxic to cells. The data obtained by the cultivation of cells on the scaffolds in vitro did not reveal the pronounced effect of scaffold composition on cell adhesion and growth. Thus, the prepared magnetoactive hybrid scaffolds of PHB/gelatin/Fe_3_O_4_ can be potentially used as magneto-responsive materials for tissue engineering, wound dressing, and controlled drug delivery using an external magnetic field.

## Figures and Tables

**Table 1:** Characteristic Raman shifts of PHB scaffolds.

Raman Shift, cm^−1^	Assignments	Raman Shift, cm^−1^	Assignments
1725	C=O stretching vibrations (crystalline phase)	1058	C–O stretching vibrations
1460	CH_3_ asymmetric bending vibrations	953	C–C stretching vibrations and CH_3_ rocking bending vibrations
1443	CH_2_ bending vibrations	841	C–COO stretching vibrations
1402	CH_3_ symmetric bending vibrations	691	C=O bending vibrations (in plane)
1365	CH bending vibrations and CH_3_ symmetric bending vibrations	680	C=O bending vibrations (out of plane)
1295	CH bending vibrations	598	C–CH_3_ and CCO bending vibrations
1261	C–O–C stretching vibrations and CH bending vibrations	510	C–CH_3_ and CCO bending vibrations
1220	COC asymmetric stretching vibrations	367	C–CH_3_ and CCO bending vibrations
1101	COC symmetric stretching vibrations	351	C–CH_3_ and CCO bending vibrations

**Table 2:** Relative atomic concentrations of the observed elements on the surface of the scaffolds and N/C ratio. Others include salt contamination corresponding to Ca, F, S, Na, and Cl.

Composite	Relative Atomic Concentration,%				N/C Ratio
	C 1s	O 1s	N 1s	Others	
PHB	74	26	–	–	–
Gelatin	66	17	15	>2	0.23
PHB/gelatin	72	20	7	>1	0.10
PHB/gelatin/Fe_3_O_4_	75	15	8	>2	0.11
PHB/gelatin (washed)	74	24	2	n/a	0.03
PHB/gelatin/Fe_3_O_4_ (washed)	74	24	2	n/a	0.03

**Table 3:** The DSC results obtained for the composite scaffolds.

Sample	T_m_, °C	ΔH_m_, J/g	X_c_,%
PHB	177.5	76.3	52.3
PHB/gelatin	169.2	24.7	16.9
PHB/gelatin/Fe_3_O_4_	167.9	13.4	9.2
gelatin	-	-	-

## Data Availability

The data presented in this study are available on request from the corresponding author.

## Supplementary Materials

The following supporting information can be downloaded at: https://www.mdpi.com/article/10.3390/polym14030529/s1, Figure S1: SEM image (**A**) and particle size distribution (**B**) of Fe_3_O_4_; Figure S2: XRD patterns (**A**) and Raman spectrum (**B**) of Fe_3_O_4_; Figure S3: Survey and high-resolution XPS spectra (**A**) of the C 1s (**B**), O 1s (**C**) and N 1s (**D**) regions for as-electrospun scaffolds and scaffolds after immersion in saline solution for 3 h.
