# Supplementary material for: Core-Shell Magnetoactive PHB/Gelatin/Magnetite Composite Electrospun Scaffolds for Biomedical Applications
Source: Polymers (Basel). 2022 Jan 28;14(3):529. doi: 10.3390/polym14030529 (PMC8839593; doi:10.3390/polym14030529)
Supplement: Supplementary file 1 [file polymers-14-00529-s001.zip › Supplementary materials.pdf]

Supplementary materials

Preparation and characterization of core-shell magnetoactive PHB/gelatin/magnetite composite electrospun scaffolds for biomedical applications

Artyom S. Pryadko[1], Vladimir V. Botvin[2], Yulia R. Mukhortova[1,2], Igor Pariy[1], Dmitriy V. Wagner[3], Pavel P. Laktionov[4], Boris P. Chelobanov[4,5], Roman V. Chernozem[1,2], Maria A. Surmeneva[1,2], A.L. Kholkin[1,2,6], and Roman A. Surmenev[1,2*]

[1] Physical materials science and composite materials center, Research School of Chemistry & Applied Biomedical Sciences, National Research Tomsk Polytechnic University, 634050, Tomsk, Russia

[2] International Research & Development Center "Piezo- and magnetoelectric materials", Research School of Chemistry & Applied Biomedical Sciences, National Research Tomsk Polytechnic University, 634050 Tomsk, Russia

[3] National Research Tomsk State University, 634050, Tomsk, Russia

[4] Institute of Chemical Biology and Fundamental Medicine, Siberian Branch, Russian Academy of Sciences, 630090 Novosibirsk, Russia

[5] Novosibirsk State University, 630090 Novosibirsk, Russia

[6] Department of Physics & CICECO - Aveiro Institute of Materials, University of Aveiro, Aveiro, Portugal

*corresponding authors: rsurmenev@mail.ru (R.A. Surmenev); kholkin@ua.pt (A.L. Kholkin)

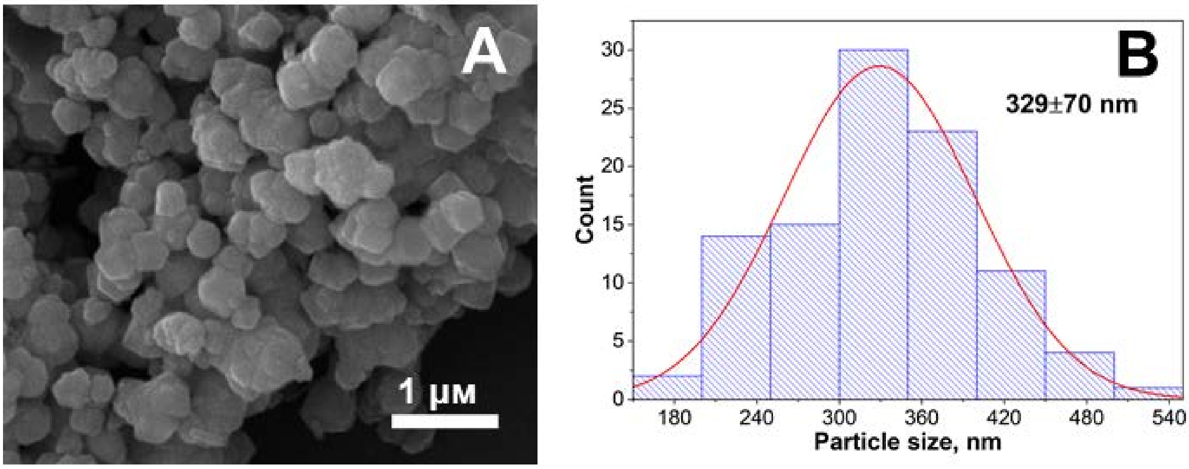

Figure S1. SEM image (A) and particle size distribution (B) of Fe_3O_4

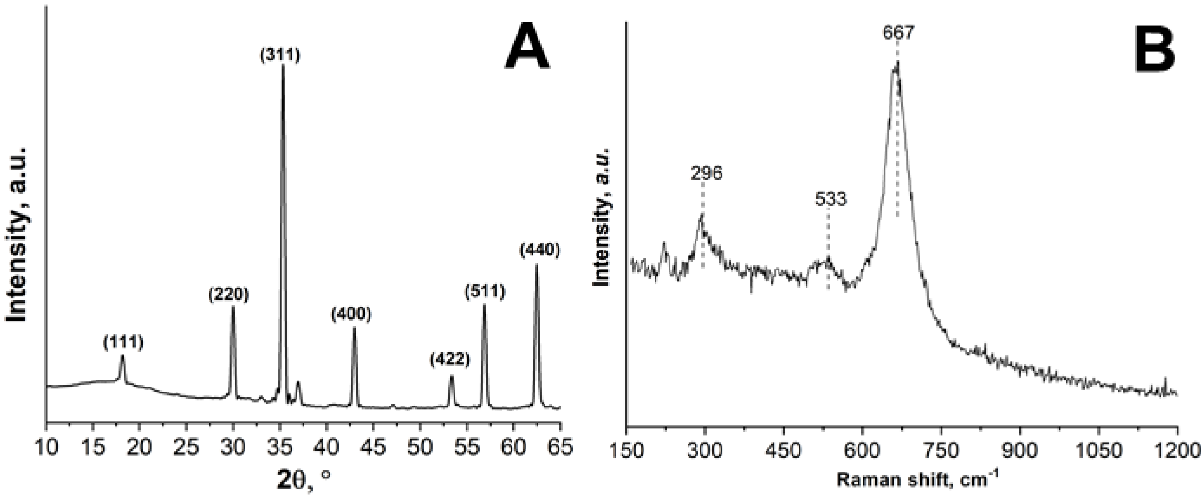

Figure S2. XRD patterns (A) and Raman spectrum (B) of Fe_3O_4

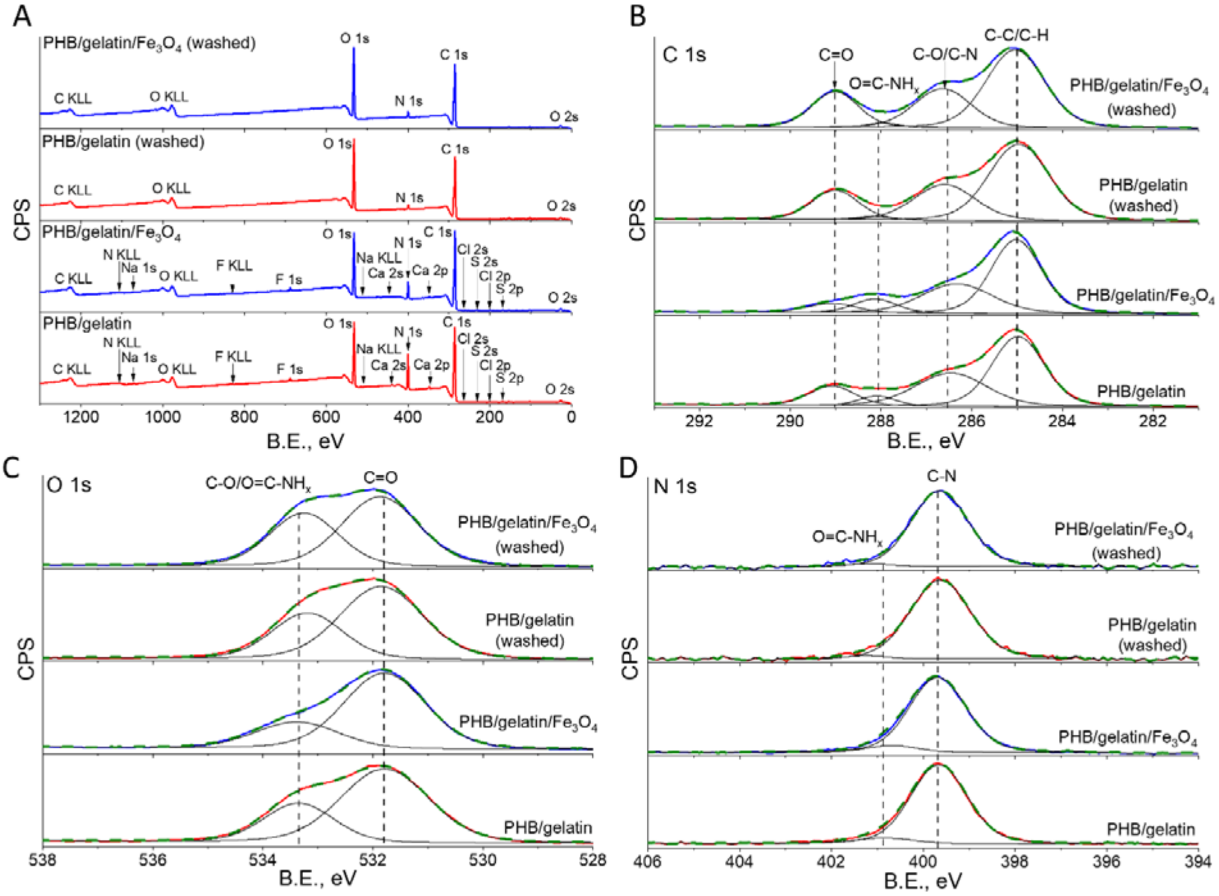

Figure S3. (A) Survey and high-resolution XPS spectra of the (B) C1s, (C) O 1s and (D) N 1s regions for as-electrospun scaffolds and scaffolds after immersion in saline solution for 3 h
